# Iron Deficiency and Iron Homeostasis in Low Birth Weight Preterm Infants: A Systematic Review

**DOI:** 10.3390/nu11051090

**Published:** 2019-05-16

**Authors:** Jorge Moreno-Fernandez, Julio J. Ochoa, Gladys O. Latunde-Dada, Javier Diaz-Castro

**Affiliations:** 1 Department of Physiology, University of Granada, 18071 Granada, Spain; jorgemf@ugr.es (J.M.-F.); javierdc@ugr.es (J.D.-C.); 2 Institute of Nutrition and Food Technology “José Mataix Verdú”, University of Granada, 18071 Granada, Spain; 3 Department of Nutritional Sciences, School of Life Course Sciences, Faculty of Life Sciences & Medicine, King’s College London, London SE1 9NH, UK; yemisi.latunde-dada@kcl.ac.uk

**Keywords:** iron, growth, development, infant, premature

## Abstract

Iron is an essential micronutrient that is involved in many functions in humans, as it plays a critical role in the growth and development of the central nervous system, among others. Premature and low birth weight infants have higher iron requirements due to increased postnatal growth compared to that of term infants and are, therefore, susceptible to a higher risk of developing iron deficiency or iron deficiency anemia. Notwithstanding, excess iron could affect organ development during the postnatal period, particularly in premature infants that have an immature and undeveloped antioxidant system. It is important, therefore, to perform a review and analyze the effects of iron status on the growth of premature infants. This is a transversal descriptive study of retrieved reports in the scientific literature by a systematic technique. PRISMA (Preferred Reporting Items for Systematic Reviews and Meta-Analyses) guidelines were adapted for the review strategy. The inclusion criteria for the studies were made using the PICO (population, intervention, comparison, outcome) model. Consequently, the systematic reviews that included studies published between 2008–2018 were evaluated based on the impact of iron status on parameters of growth and development in preterm infants.

## 1. Introduction

Iron is involved in many key cellular functions and processes in humans due to its role as an essential micronutrient. This ubiquitous mineral plays a critical role in growth and the development of the central nervous system. It is essential for energy metabolism, cell differentiation, and a host range of physiological processes for the normal functioning of the brain [1]. In spite of this, iron deficiency (ID) is the most prevalent micronutrient deficiency in the world, and it affects all age groups, children between 0 and 5 years of age being the most affected [2]. Therefore, iron requirements are particularly high during the periods of rapid growth and differentiation and imbalance in iron homeostasis could result in alterations of cognitive functions and neurodevelopment [3].

Preterm infants are prone to develop iron deficiency anemia (IDA) in the first 4 months of life due to lower iron stores at birth compared with term infants, rapid growth and iron losses. Most fetal iron is transferred from the mother during the third trimester of gestation. This transfer is interrupted by preterm birth, resulting in iron stores at birth being proportional to birth weight. Despite low iron stores at birth, growth velocity of premature infants is maximal at a postmenstrual age of 28–38 weeks, reflecting a particularly high iron need. The risk of IDA in preterm infants is further increased by frequent uncompensated iatrogenic phlebotomy losses [3,4,5]. 

Initial evaluation should include a clinical history, to assess prematurity, low birth weight, diet, chronic diseases, family history of anemia, and ethnic background. A complete blood count is the most common initial diagnostic test used to evaluate for IDA, and this count allows for differentiating microcytic, normocytic, and macrocytic anemia based on the mean corpuscular volume (MCV). Ferritin measurement is the most sensitive test for diagnosing IDA, being a good reflection of total iron storage and is also the first laboratory index to decline with ID [6].

Developmental outcomes are positively influenced by adequate nutrition early in life, revealing an improvement in neurodevelopment and cognitive abilities in preterm infants, for instance. During development, ID negatively affects the growth and functioning of multiple organ systems particularly the brain, skeletal muscle, heart and the gastrointestinal tract [7,8,9,10,11]. IDA in preterm infants could induce impaired cell differentiation [12,13] and alter normal neurodevelopment processes [14,15]. Due to increased risk of developing IDA in premature infants, iron supplementation is recommended in some situations. Although iron supplementation improves iron status of preterm infants [16], the nature and the means by which this is achieved are not clearly defined [17,18]. Preterm infants do not have a fully developed antioxidant capacity and free iron acts as potential oxidative stressor, iron supplementation, therefore, has to be considered cautiously in this population [4].

An excess of iron can affect organ development during the postnatal period. There is an association between iron overload, retinopathy of prematurity, and bronchopulmonary dysplasia. Non-protein bound iron mediates oxidative stress in the presence of poor antioxidant capabilities, and this condition initiates or potentiates the progression of these pathologies through generation of free radicals [4]. ID and toxicity could be avoided by an adequate iron homeostasis, which is essential for optimal development and function. 

There is a high prevalence of preterm babies who have an increased risk of IDA, and there are no clinical consensuses or guidelines about the clinical benefits of iron supplementation and/or fortification that the hospitals should follow with this extremely vulnerable population. Therefore, it is relevant to evaluate the effects of ID or iron toxicity on the optimal development and function of the organs such as the brain in premature infants, ID and iron overload disorders during this period [4].

Consequently, this systematic review performed an analysis of the influence and effects of iron on the hematological parameters, growth, and development of premature infants. Thus, the review also revealed a systematic literature evidence on the beneficial effects of iron supplementation in premature infants.

## 2. Materials and Methods

### 2.1. Design

Descriptive study of the articles retrieved using a systematic technique.

### 2.2. Sources of Data Extraction

The data used in this study were obtained from the consultation and direct access to the scientific literature collection in the following databases: Medlars International Literature Online (MEDLINE) via PubMed (USA National Library of Medicine National Institutes of Health Search database), The Cochrane Library Plus, SCOPUS (World’s largest abstract and citation database of peer-reviewed literature), Web of Science and Literatura Latinoamericana y del Caribe en Ciencias de la Salud (LILACS).

### 2.3. Search Strategy

A search strategy for electronic databases was developed with regard to information processing, from the Thesaurus study, Medical Subject Headings (MeSH) developed by the U.S. National Library of Medicine.

The use of the following terms was considered appropriate: “Iron”, “Growth and Development”, and “Infant, Premature”, as well as descriptors as text in the title and summary record fields. A search equation was developed for use in the MEDLINE database, via PubMed, using boolean connectors, and then adapted to the rest of aforementioned data bases: (“Iron”(Mesh) AND “Growth and Development”(Mesh) AND “Infant, Premature”(Mesh)). The search was expanded to include “Anemia, Iron-Deficiency” (Mesh).

### 2.4. Eligibility Criteria

The proposed criteria for the studies included in the review, considering the PICO (population, intervention, comparison, outcome) model [19] were: (a) studies published in peer-reviewed journals; (b) studies involving infants who were premature (gestational age <37 weeks) or with a low birth weight (<2500 g); they are especially susceptible to developing IDA because these infants have smaller iron stores at birth and a greater iron requirement concurrent with a rapid increase in the red cell mass; (c) studies had to inform quantitatively about the iron status and iron supplementation.

Studies were excluded if the full text was not found, if they were not carried out in humans, if the premature infants presented diseases different to anemia, if they were identified as redundant publications, if they were not written in English or Spanish, or if they did not include an empirical result related to the iron status in the organism of premature infants and their influence on growth and development. We also limited references to the last 10 years (2008–2018), as the calculated period of obsolescence (half period of Burton Kebler [20]).

A multidisciplinary research team developed the search, inclusion and exclusion criteria. Pediatrics and nutritionist research experts also took part in this process. Furthermore, a biostatistician was involved in this phase.

### 2.5. Study Selection

The initial phase of criteria selection was the exclusion of duplicates. Articles whose titles and/or abstracts were not clearly related to the subject of study were also excluded. Once all potentially eligible articles had been located, each of them was carefully reviewed to establish further eligibility criteria and all relevant information and data were extracted. 

The selection of the articles was performed independently by two authors: J.M-F and J.D-C, and it was established that the valuation of the concordance between the authors (Kappa index) should be greater than 80% [21]. In the case of a discrepancy, the authors reviewing the literature made a consensus decision. Additionally, the reference list of every selected article was carefully checked to identify other potentially eligible articles, which were processed in the same way as those retrieved from the electronic search. Independently, both reviewers read the full text of the remaining articles and screened for inclusion. Afterwards, they compared the results of the full text screening. A predefined template was used to perform data extraction from eligible articles. 

### 2.6. Data Extraction

Two independent reviewers extracted the data. To increase uniformity and reduce bias, a standardized data collection form was used by each reviewer. The reviewers made a consensus decision when there was a discrepancy. From each eligible study, the following information/data were extracted: first author, year of publication, journal, eligibility criteria, definition of premature or low-birth weight, number of participants, hematological data (hemoglobin (Hb), hematocrit (Ht), serum iron) before iron supplementation, iron supplement dose, duration of iron supplementation, hematological data after iron supplementation, growth status after iron supplementation, adverse effects of iron supplementation, and effects of iron on cognitive status.

### 2.7. Risk of Bias in Individual Studies

The final sample of studies for review was subsequently assessed by the authors (Table 1) separately to convey the methodological and critical appraisal using the Delphi List appraisal tool [22] for randomized studies. This tool is composed of an eight item scale, including questions that are rated according to yes/no/don’t know. A response of “yes” is indicated by the numeric score of 1 and a response of “no” or “don’t know” is indicated by a 0 (with a maximum score of 8). The Delphi List achieved consensus on a generic criteria list for quality assessment in randomized clinical trials. The adoption of this core set is the minimum reference standard of quality measures for all randomized clinical trials, where a score of 1 means high risk of bias and 8 means a low risk. 

Non-randomized studies were assessed using the Transparent Reporting of Evaluations with Non-randomized Designs (TREND) statement [23], which contains a list of 22 essential aspects (available at http://www.TREND-statement.org) that must be described in the publication. The TREND statement is specifically developed to guide standardized reporting of nonrandomized controlled trials. The TREND statement complements the widely adopted CONsolidated Standards Of Reporting Trials (CONSORT) statement developed for randomized controlled trials. This is a collective effort in promoting the idea that transparent reporting is valuable to improve research synthesis and advance evidence-based recommendations for best practices and policies. A point was assigned for each item featured. Meanwhile, if an item was not applicable, it did not score (a maximum score of 22). When an item was composed of several points, these points were evaluated independently, giving the same value to each of them, and subsequently an average was made (being the final result of each item), in such a way that in no case the score of one point per item could be exceeded. For each included study, a consensus was achieved after discussion of discrepancies. Table 1 and Table 2 summarize the score for each study and criterion included in the Delphi List and the TREND statement. Based on the quality appraisal, no studies were discarded. 

Upon thoroughly reviewing the data collection and statistical analysis of each included article, it became apparent that there was too much variation between these studies, and, therefore, it was not considered appropriate to conduct additional analysis such as a meta-analysis using the reported findings.

J.M-F and J.D-C reviewed the articles independently, extracted relevant data from each included article, and assessed risk of bias. The Delphi List was carried out in the 5 randomized studies selected, while the TREND Checklist was used in the 9 non-randomized selected manuscripts. Any discrepancies in scoring were resolved by mutual discussion or through discussion with J.J.O. An overall risk of bias rating was assigned to each study based on the quality score.

The Delphi List carried out in the selected manuscripts is provided in Table 1. The total score for each report is shown in the last file constituting the sum of scores for each criterion. The maximum possible score was 8, but this was not assigned to any manuscript. Reports ranged between 5 [24,25] and 7 [26,27,28,29], with an average score of 6.2. The TREND Statement Checklist is provided in Table 2. The total score for each report is shown in the last file constituting the sum of scores for each criterion. The maximum possible score was 22 but was not assigned to any manuscript. Manuscripts ranged between 10.92 and 13.85, with an average score of 12.19.

## 3. Results

### 3.1. Study Selection

The above methodology resulted in a total description of search criteria and provided a total of 108 citations. The title and abstract of each report were reviewed by J.M.-F. and J.D.-C. to assess eligibility, and if the abstract or title was not described in enough detail, full reports were assessed to consider their inclusion. In this sense, all full-text reports for inclusion eligibility were independently assessed by J.M.-F. and J.D.-C. The level of agreement between the two researchers was high enough. The researchers resolved minor differences related to the inclusion of the reports by discussion. The potential lists of each relevant recovered article were scanned and four more articles were identified as possibly relevant. 

**Figure 1:**
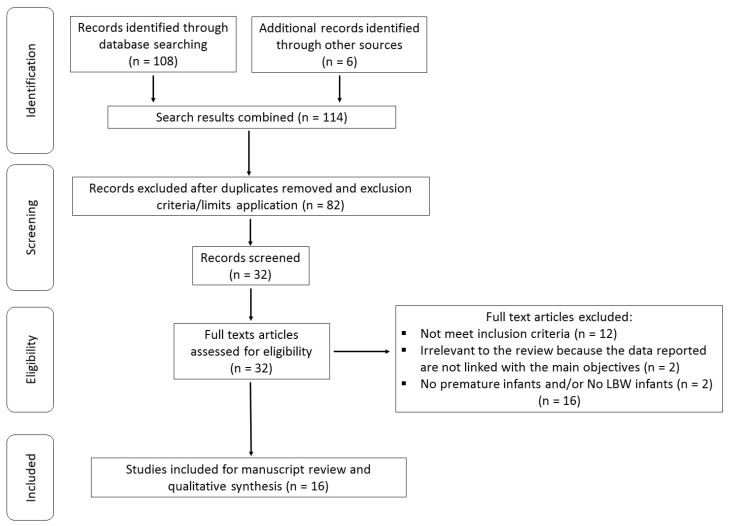
Flowchart showing study selection procedure and results.

Duplicate manuscripts and those based on exclusion criteria and limits were removed. Thirty studies remained potentially eligible (Figure 1). Sixteen studies were excluded because they did not meet the inclusion criteria. Finally, 16 studies met all inclusion criteria and were incorporated in the current review. 

### 3.2. Study Characteristics

The summary of the most relevant characteristics of the randomized and non-randomized studies included are shown in Table 3 and Table 4, respectively. Out of the 16 studies included, four were conducted in India [24,25,31,35], three were carried out in the Netherlands [30,32,33], four were performed in Sweden [26,27,28,29], three in the USA [36,38,39], one in Japan [37], and one in Brazil [34]; these studies were published between 2009 and 2017.

Regarding the randomization, this review includes six randomized studies [24,25,26,27,28,29] (being [26,27,28,29] results from the same original cohort) and 10 non-randomized studies [30,31,32,33,34,35,36,37,38,39]. Four of these studies were double-blind [26,27,28,29].

Sample sizes ranged from 46 to 401 subjects. Among the total of 1743 participants included in the trials, 50.95% were men and 49.05% were women. One study did not specify the number of male and female participants [27]. The age preterm participants in the study ranged from 24 to 36 weeks. Yamada and Leone [34] included term newborns at 37 to 41 weeks of age. None of the included participants featured other pathologies apart from anemia. Most of the studies used iron supplements (specifically nine reports [24,25,26,27,28,29,34,36,38]) and the remaining studies did not use iron supplements [31,32,33,35,37,39]. 

Among all included studies, a variety of different iron status parameters were investigated. Most of the studies analyzed Hb, serum ferritin (SF), serum iron, and total iron-binding capacity (TIBC). Neurological development was also studied to measure the developmental assessment scale and child behavior checklist questionnaire. Berglund et al. [28] followed the participants from birth to 3.5 years of age and performed the Wechsler Preschool and Primary Scale of Intelligence (WPPSI-III) to assess cognitive function. The auditory brainstem response (ABR) was assessed as an indicator of the development of the central nervous system (CNS) [27,39], and a Developmental Assessment Scale for Indian Infants (DASII) was also used to evaluate neurodevelopment [25]. Moreover, Berglund 2018 tested children at 7 years of age for psychometric intelligence quotient (IQ) using the validated Wechsler Intelligence Scale for Children (fourth edition, WISC-IV), Child Behavior Checklist (CBCL) questionnaire, and Five to Fifteen (FTF) test. 

### 3.3. Characteristics of Iron Status Measurements

All of the studies included in the review assessed the iron status in preterm neonates to evaluate iron metabolism and requirements, supplying a large number of different measures. SF levels were reported in all the studies except Amin et al. [39], who measured cord ferritin. The SF level reflects total body iron stores, providing a convenient method of assessing the status of iron storage. SF is measured as a routine biomarker, although it is now known that many additional factors, including inflammation, infection, and malignancy complicate the interpretation of this value. Nine studies reported Hb levels [24,26,27,28,29,30,31,32,33,34] (Hb is a heterotetrameric protein that reversibly binds oxygen. Hb transports heme-bound oxygen from the lungs to all of the tissues in the body via oxygenation-linked shifts in the conformational equilibrium between the tense state and the relaxed state). This structural transition is controlled allosterically, as the binding of oxygen to one subunit affects the oxygen-affinity of the other subunits in the same tetrameric assembly), four measured Ht levels [24,31,34,38], four measured serum iron levels [26,29,34,35,38] (Ht is expressed as red blood cells packed cell volume, which is a very useful clinical factor in hemodialysis, surgical procedures, and anemia, which can be used to estimate transfusions and determine the extent of anemia), five measured transferrin saturation levels [26,27,28,29,34,37] (the transferrin saturation is an index that takes into account both plasma iron and its main transport protein and is considered an important biochemical marker of body iron status), three assayed TIBC levels [34,35,38] (TIBC indicates the maximum amount of iron necessary to saturate all available transferrin iron-binding sites, therefore, it correlates well with transferrin concentration), and three studies analyzed transferrin receptors [26,27,28] (the transferrin receptor mediates cellular iron uptake through clathrin-dependent endocytosis of iron-loaded transferrin, playing a key role in iron homeostasis).

Two studies measured reticulocytes counts [30,34], one study reported transferrin saturation [26,29], one manuscript revealed percentage of total iron binding capacity [38], and one study assessed red cell counts [37]. Moreover, one study incorporated Zinc protoporphyrin/heme (ZnPP/H) levels as a possible iron status indicator [30]. Berglund et al. [28] also measured hepcidin concentration. In addition, another study reported a serum prohepcidin concentration [37].

### 3.4. Iron Status and Body Composition

In all studies that analyzed the weight, the length and head circumference of premature infants was lower than that of the term infants. As suspected, these measurements were lower in preterm infants when compared to term infants, although Yamada and Leone [34] showed an increase in weight, length, head circumference, and BMI (*p* < 0.001) at an expected rate for the age of the population during the postnatal development of all infants, although these measurements were lower in preterm infants. The infants with iron depletion showed a lower birth weight than those without iron depletion. Lower birth weight and SF concentrations are independently associated with iron depletion at 6 weeks of age [32]. A study conducted by de Waal et al. [30] showed a decrease of iron available for erythropoiesis, when birth weight had already doubled at 6 weeks, and a subsequent increase of ZnPP/H levels. However, these results should be interpreted with caution and take into account the weak study design and subsequent high Risk of Bias (RoB). With regard to iron supplementation, mean birth weight was lower in infants who received supplementation as compared with those who did not [33]. On the other hand, weights of infants at 2 months were not different between the intervention and control groups [24].

In addition, a lower birth weight was found in infants born with iron overload compared to those with normal iron status and latent ID status (serum ferritin ˂76 ng/mL or ˂170.7 pmol/L) [36]. Other studies did not find differences between iron supplemented groups and controls in mean weight, length, head circumference, or knee heel length at 6 months [26].

### 3.5. Iron Deficiency

ID was evaluated despite the different iron status indices used in the studies consulted. Most of the studies revealed that preterm or late preterm neonates usually featured a high risk of ID. 

In this sense, indicators of iron status, including Hb levels and the prevalence rates of ID and IDA at 6 months, differed significantly in a dose-dependent manner between the intervention groups. Berglund et al. [26] found that healthy marginally low birth weight infants, both preterm and term, had high risks of developing ID (36%) and IDA (9.9%) at 6 months, especially if they were exclusively breastfed at 6 weeks (ID, 56%; IDA, 18%). This finding is in agreement with the study developed by Uijterschout et al. [33], which reported that ID was present in 18.9% and 4.9% of preterm infants at the age of 4 and 6 months, respectively. IDA was present in 7.7% at the age of 4 months and in 1.4% of preterm infants at the age 6 months. Akkermans et al. [32] showed that iron depletion and iron-depleted anemia in preterm infants at 6 weeks were present at 38.2% and 30.9%, respectively. They showed that preterm infants with a birth weight <1830 g and a SF concentration of <155 μg/L in the first week had a 26.4 times higher risk to develop iron depletion compared to infants with normal birth weights. Similarly, Mukhopadhyay et al. [35] developed a study comparing preterm infants who were small for gestational age, preterm infants appropriate for gestation, and term infants appropriate for gestation. These studies showed that iron stores were significantly decreased in small, preterm, gestational age infants compared to preterm infants appropriate for gestation at birth and at 4 weeks. Likewise, term infants appropriate for gestation had higher iron stores compared to preterm infants appropriate for gestation. In their study, the extent of infants with SF ≤35 μg/L was significantly higher in preterm infants who were small for their gestational age compared to the preterm appropriate for gestation group (*p* = 0.01). Moreover, ferritin levels were significantly lower (*p* = 0.006) those in the preterm appropriate for gestation group compared to the term appropriate for gestation infants. Serum iron also decreased in the preterm small for gestational age group compared to the preterm appropriate for gestation group (*p* = 0.002). Amin et al. [39] showed that 44% of preterm infants had ID (cord ferritin less than 75 ng/mL) and 56% had normal iron status (cord ferritin higher than 75 ng/mL). Once more, these results could be lack of strength to account for the low score obtained in the TREND List and subsequently high RoB.

In this sense, Yamada and Leone [34], indicated that the incidence of anemia was 66.67% in late-preterm newborns at a gestational age of one month post-term and 41.18% in term newborns at one month of age (*p* = 0.02). Hb and Ht decreased mainly in the late-preterm newborns (*p* < 0.001). At birth, differences between the two groups were observed only for Ht (*p* = 0.010), whereas at two months, more dramatic differences in both Hb (*p* = 0.020) and Ht (*p* < 0.001) were reported. Iron concentrations in the late-preterm infants were lower at term (*p* = 0.0034) and at one month post term (*p* < 0.001). TIBC showed lower levels when measured in the late-preterm infants at birth (*p* < 0.001). Transferrin saturation presented a similar pattern and was lower in late-preterm newborns at birth (*p* < 0.001). In a study conducted by de Waal et al. [30] with premature infants at 4 months of age, ID was present in 21.3% and IDA in 8.5% of cases. ZnPP/H levels were used as a possible indicator of iron stores, and at 4 months of age, infants with IDA showed higher levels compared with normal infants (*p* < 0.001). At the same time point, infant with ID did not show any differences in ZnPP/H levels compared with normal infants *p* = 0.223). In addition, a study conducted by Amin et al. [36] revealed that of 131 premature infants, 23% had a latent ID status, 58% had normal iron status while 19% had iron overload status. 

Conversely, others studies reported that there were no significant differences between preterm or term infants with regards to ID. Therefore, Molloy et al. [38] observed that all the iron indices examined in preterm infants were elevated above standard normal values in these infants. Sankar et al. [24] described a study with very low birth weight (VLBW) preterm infants, and they showed that the SF at 60 days was not different between VLBW and control groups (full term infants) as well as the mean Ht or Hb at 60 days that were not different for the two groups. Kitajima et al. [37] observed that preterm and full-term infants became anemic during the neonatal period (*p* = 0.005, *p* = 0.018, respectively). At the birth of preterm infants, SF levels were lower than those of full-term infants (*p* = 0.011), and the transferrin saturation of preterm infants did not differ between birth and 1 month after birth levels. Preterm infants showed a significant increase in prohepcidin levels (*p* = 0.011), a finding not shown in full-term infants.

### 3.6. Iron Supplementation

Iron supplementation was established in 9 studies. Most of the studies supplied iron as elemental iron in ferrous sulfate chemical form, colloidal iron and ferrous succinate mixture while 3 studies did not specify the chemical form of iron supplement [31,34,38]. The differences in iron dosage used in the studies ranged from 1 to 4 mg/kg/day. In addition, the supplementation period started after one or two months of age, but in the majority of the studies, the duration of the supplementation period was not reported. Only four studies specified exactly this period, from 6 weeks to 6 months age [26,27,28,29].

### 3.7. Effects of Iron Supplementation

In the scientific literature, controversies abound on the necessity of t iron supplementation. Some studies showed a positive effect of iron supplementation and its role in preventing or treating ID. In a study with marginally low birth weight preterm or term infants, Berglund et al. [26] showed that iron supplements at a dose of 2 mg/kg per day from 6 weeks to 6 months reduced the risk of IDA effectively, without adverse effects on morbidity or growth observed during the period of supplementation. Taking into account the high score obtained in the Delphi list, and the subsequently low RoB, this dosage and period of supplementation should be considered more plausible than the results reported by other studies. Akkermans et al. [32] revealed that early individualized iron supplementation should be considered as a therapy for high-risk infants with a birth weight of <1830 g and an SF in the first week of <155 μg/L, because late preterm infants have a 26.4 times higher risk of developing iron depletion. In this way, Yamada and Leone [34] suggested the necessity of specific iron supplementation in breastfed late preterm newborns at one month post-term, because greater reductions in Hb/Ht and lower iron stores were found in the preterm infants compared with term newborns.

In contrast, other studies did not show any effect of iron supplementation. Saha et al. [31] suggested that late preterm and term infants (small for gestational age) have adequate iron stores at birth and at 2 months of age; therefore, they do not need iron supplementation until at least the second month of life. In this way, Sankar et al. [24] did not find any difference in SF at 2 months of age between the iron supplementation and control groups when 3 or 4 mg/kg/day (during 2 weeks) of iron supplementation were supplied at 2 weeks of life. In addition, Molloy et al. [38] found elevated iron indices in premature infants prior to establishing iron supplementation. In particular, male premature infants were more susceptible to increased iron levels despite similar numbers of blood transfusions, gestational age, birth weight, albumin, and direct bilirubin. Therefore, iron supplementation should be avoided to prevent iron overload. On the other hand, Amin et al. [36] showed that enteral iron supplementation is unlikely to cause iron overload in premature infants due to the fact that all subjects received enteral iron supplementation of ~2 mg/kg/day, and there was no difference in post-menstrual age when infants reached full volume of enteral feeding (or when iron supplementation was initiated). 

Uijterschout et al. [33] proposed that individualized iron supplementation as an alternative to established supplementation therapies be further investigated, and ferritin should be used as a measurement to individualize iron supplementation to late preterm infants. However, it needs to be taken into account that the measurement of ferritin as a sole biomarker may not be appropriate because ferritin is an acute phase protein; therefore, it will not be accurately reflected in the state of inflammation.

### 3.8. Iron Status and Cognitive Performance

Of the studies included in this systematic review, three directly investigated the effects of iron status on cognitive performance in premature infants. The techniques used to assess cognitive performance were noninvasive. 

Berglund et al. [27] tested the auditory brainstem response (ABR) system, which is an auditory evoked potential extracted from ongoing electrical activity in the brain and recorded via electrodes placed on the scalp. This is a neurophysiological method for assessing the development of the central nervous system (CNS) in infants of a marginally low birth weight. They did not report significant differences in absolute ABR latencies between groups, even though the iron supplementation resulted in a significant difference in the prevalence of ID and IDA. This result is surprising taking into account that prolonged latencies were caused by delayed CNS myelination in infants with early IDA. Therefore, they did not find positive or adverse effects of iron supplementation (1–2 mg/kg/day of iron supplementation), on absolute wave I or V latencies at 6 months of age in marginally low birth weight infants, even though they are at risk for ID and IDA. Another study described by Amin et al. [39], revealed that infants with normal iron status had a higher ABR response compared to the ID group (*p* = 0.08).

Another study by Berglund et al. [28], used the Wechsler Preschool and Primary Scale of Intelligence (WPPSI-III) to evaluate cognitive functions in control and premature infants given iron supplementation (1 or 2 mg/kg/day) or placebo (0 mg/kg/day). This study aimed to evaluate the cognitive function and revealed no significant differences between the groups after 3.5 year of the intervention. In addition, in this study, parents were asked to complete the Child Behavior Checklist (CBCL) questionnaire (a widely used caregiver report form that identifies problem behaviors in children and provides a meter stick for measuring whether amounts of behavioral problems have changed over time or across societies and is a complement to other approaches for looking at rates of mental-health issues), before the 3.5-year visit at the study center, in order to assess various types of behavioral and emotional problems. The total CBCL scores revealed that there was a significantly higher prevalence of children above the US subclinical cutoff and above the 90th percentile of the Swedish reference group (Swedish cutoff) in the group who did not receive iron supplementation compared with control subjects and compared with the iron-supplemented groups (1 or 2 mg/kg/day). There was a significant increase in the iron supplemented groups compared to placebo in the CBCL “emotionally reactive” (*p* = 0.040) and “attention problems” (*p* = 0.022) subscales and a similar trend in all subscales except “withdrawn.” This study was reported to be the first showing a positive effect of early iron supplementation on neurobehavioral development beyond 2 years of age. This is another surprising result because ID impairs brain development and interferes with neurotransmitter function and biosynthesis, particularly with regard to dopamine and other monoamines. 

Berglund et al. [29] tested a psychometric IQ on children 7 years of age using the validated WISC-IV, performed by an experienced authorized pediatric psychologist. Moreover, in this study they also developed a CBCL questionnaire and FTF test (the intervention was performed on LBW infants). These tests assessed behavioral, cognitive, and emotional performance and revealed no significant differences between ID and non-ID groups.

In general the results observed in the different studies of Berglund [26,27,28,29] have stronger study designs than the other reports assessing iron status and cognitive performance due to the high scores obtained in the Delphi list, and the subsequently low RoB.

Gupta et al. [25] evaluated neurodevelopment (by a single certified clinical psychologist using the developmental assessment scale) in premature infants who received complementary feeding at 4 months or continued milk feeding and initiated complementary feeding at 6 months (supplemented with iron at 2-3 mg/kg per day). The motor and mental development quotients in the two groups were similar and did not show significant differences between the two groups. 

## 4. Discussion

This review investigated the literature available about the iron status of premature infants and their growth and development. Moreover, it evaluated the effect of iron supplementation in this age group. Premature infants revealed a lower weight, length, and head circumference compared to term infants. The preterm infants with iron depletion had a lower birth weight than those without iron depletion. Iron depletion at 6 weeks of age was independently associated with lower birth weights together with lower SF concentrations [32]. Low iron stores are significantly influenced by low birth weights in preterm infants in general and are more specific in premature infants born after <32 weeks of gestation [40,41]. An impaired placental function caused by pregnancy-induced hypertension, maternal smoking, or gestational diabetes could result in low birth weight [40]. The placenta regulates maternal-foetal iron transport during gestation to supply this essential nutrient to the foetus. Akkermans et al. [32] stated that the most important independent risk factor for iron depletion and iron-depleted anemia in late preterm infants at the age of 6 weeks is a lower birth weight, because they did not find an association between the impaired placental function and depleted iron stores adjusted for birth weight.

### 4.1. Iron Status

Regarding iron status, results from the studies analyzed showed the absence of consensus on how to define iron stores in preterm infants. SF measurement is the most widely used index to evaluate iron status and it was measured in all studies. Even so, SF is not considered the best iron status parameter by all the authors, because it changes over time and increases during infection and inflammation [30,33,34,42,43]. Therefore, additional biomarkers such as ZnPP/H ratios, which are an indicator of iron available for erythropoiesis, have been proposed [44]. ZnPP/H levels should increase after birth, representing higher iron needs due to rapid growth and iatrogenic iron losses, but should decrease in the first 6 weeks postnatal and return to baseline levels after this period. In premature infants with ID, ferritin levels were low, but ZnPP/H levels were high, showing no association between both parameters. There is a difference between the iron parameters referred to by ferritin and ZnPP/H [30]. Stored iron in the body is reflected by ferritin levels, whereas iron available for erythropoiesis is directly related to the ZnPP/H ratio [30]. In addition to the SF assessment, other traditional iron status parameters such as Hb, Ht, serum iron, transferrin, transferrin saturation, transferrin receptor, and TIBC were evaluated. Most of the data revealed that preterm or late preterm neonates usually showed a lower iron status than term neonates. Yamada and Leone [34] revealed that Hb and Ht significantly reduced while reticulocytes increased during all assessments in the late preterm infants, which might indicate the presence of intense bone marrow stimulation for erythropoiesis. After birth, ferritin levels decreased until two months of age in term and preterm infants [34]. Between 25% and 85% of preterm infants develop evidence of iron deficiency during infancy [34]. Unlike full term infants, in whom the condition typically occurs during the second half of infancy, preterm infants are at risk of developing iron deficiency during their first postnatal months [45]. Iron deficiency is more common in preterm infants from developing countries and in those consuming human milk exclusively without supplementation [46]. A number of factors combine to predispose the premature infants to a negative iron balance. Iron is mostly accumulated during the third trimester of gestation. Total body iron and Hb contents as well as serum and storage iron concentrations are lower in preterm infants. Conditions such as severe maternal iron deficiency, intrauterine growth restriction [47], and chronic blood loss during gestation can further compromise fetal iron endowment.

Mukhopadhyay et al. [35] used ferritin, serum iron, and TIBC as a combination of various biomarkers to assess the iron stores, and they found that preterm infants who were small for their gestational age presented significantly fewer iron stores at birth and at 4 weeks of age compared to preterm infants appropriate for gestation. Low SF levels could be explained by the loss of iron from the storage pool due to chronic hypoxia and increased red cell mass, together with a smaller size of the placenta, which implies lesser surface area, or due to placental vascular disease reducing the transport.

Iron has a crucial role in many metabolic pathways, especially in erythropoiesis and neurodevelopment [1]. Specifically, Hb formation, which is crucial for oxygen transport to tissues, is dependent on iron absorbed from the diet and storage. If the absorption of dietary iron is insufficient or the deposits are depleted, Hb still can be synthesized until serum iron decreases. After birth, higher environmental oxygen concentrations, a decrease in fetal Hb, an increase in adult-type Hb, polycythemic conditions, and Hb oxygen saturation promote great tissue oxygenation, thereby decreasing the stimulation of erythropoietin production and reducing erythrocyte release. Therefore, Hb levels are reduced by 30% to 50%, reaching their minimum value at six to twelve weeks after birth and one to four weeks earlier in preterm infants [48]. Furthermore, the fetal iron storage of preterm infants is incomplete before their birth, increasing the risk of ID and even IDA during the early postnatal period [41,49,50].These results suggest that preterm infants have increased iron needs after birth [34]. In addition, irreversible processes such as impaired neurological development in preterm infants may be caused by ID, even without anemia [51].

Although, some studies did not find lower iron parameters in premature infants after birth, Molloy et al. [38] found that in preterm infants some iron indices were elevated above the standard normal values in these infants. In this way, Saha et al. [31] observed that the Hb and SF of all late preterm infants were within a normal range at 2 months of age, which implies adequate stores at birth to meet requirements. The inflammation/infection process could explain these levels, which has been associated with an increase of SF.

Taking into account iron status in preterm infants, it seems highly relevant to study the presence or absence of ID or IDA in this population. Premature infants have increased risk to develop ID, which is the most common micronutrient deficiency in the world [52], and also IDA, due to low endowment of iron stores, long periods of parenteral nutrition without iron supplementation, phlebotomy loss, and rapid catch-up growth and development [4]. Most of the selected studies revealed that ID and IDA were present in premature infants, being more common when the premature infants were exclusively breastfed [26,28,53]. Moreover, ID and IDA have been associated with long lasting abnormal neurodevelopmental outcomes in full-term, normal weight infants, during the critical period of brain development [1,54], due to their effect on cognitive and psychomotor development, even after iron treatment [55]. In addition, growth and functioning of other organ systems such as skeletal muscle, the heart, and the gastrointestinal tract could be also affected by ID [8,10].

Dewey et al. [56] reported that iron supplementation (1 mg/(kg/day)) to breastfed infants may benefit infants with low hemoglobin concentrations but may present adverse effects, such as deficits in gains in length and head circumference and an increased risk of diarrhea for those with normal hemoglobin concentrations. Majumdar et al. [57] similarly found that iron supplementation (2 ng/(kg/day)) benefited iron-deplete children aged 6–24 months but led to deficits in weight and linear weight gain in those who were iron replete. 

### 4.2. Iron Supplementation

According to these results and taking into account the importance of avoiding ID in this population, iron supplementation of preterm neonates seems vital because rapidly depleting inadequate iron stores cannot meet the iron requirement of enhanced erythropoiesis during this period. However, previous studies on iron supplementation in preterm infants have yielded conflicting results [45,58]. Although iron is essential for children’s development, iron overload should be considered because of the potential risk of iron excess and the poorly developed antioxidant mechanisms in preterm infants, facts that reinforce the idea of avoiding indiscriminate iron supplementation in this population [4,59]. Iron overload mediates oxidative stress and may also contribute to free oxygen radical injuries that are typical of premature infants, such as chronic lung disease and retinopathy from prematurity [60,61]. IDA was reduced effectively with a dose of 2 mg/kg per day from 6 weeks to 6 months [26], and non-adverse effects on morbidity or growth were found. In addition, as most pathogenic organisms need iron as an essential nutrient, it has been suggested that iron supplementation may increase the risk of infections, but in this study, the supplementation did not affect infections or other pathological symptoms. Moreover, another study suggests that iron supplementation is justified in late preterm infants with a birth weight of <1830 g and ferritin concentrations of <155 μg/L in the first week [32]. 

In contrast, Sankar et al. [24] did not find any association with iron supplementation at 2 weeks of life, improving neither SF nor hematological parameters at 2 months of age at preterm. This could possibly be due to inadequate iron absorption, inappropriate dosage or a “contamination” effect (iron preparation used in this study contains colloidal iron in the form of non-ionic, microfine particles). By the second month of life, erythropoiesis is increased and iron stores are rapidly utilized. Although SF levels decreased after birth due to the utilization of iron stores, both the SF and Hb of all premature infants were within normal range at 2 months of age [31]. Therefore, iron stores at birth were adequate to meet the requirements of these infants for at least 2 months of life, and iron supplementation is not necessary during this period. In this sense, Molloy et al. [38] found elevated iron indices in infants prior to commencing iron supplements, so an evaluation of iron stores after birth is essential to prevent potential iron overload and unnecessary iron supplementation. However, another explanation could be unmeasured confounding factors in these studies.

### 4.3. Cognitive Performance

Regarding the effect of iron supplementation on cognitive performance, Berglund et al. [27] evaluated the effect of 1–2 mg/kg/day of iron supplementation on ABR as a noninvasive, objective, neurophysiologic method for assessing the development of the central nervous system. ABR latencies measure the conduction speed in the auditory system from the cochlea to the inferior colliculus in the upper brainstem. From late gestation through the first 3–6 months of life, these latencies experience a rapid decrease continuing at a slower rate during the first 3–5 years of life, indicator of CNS myelination during infancy for preterm and term infants. In this study, no significant positive or adverse effects in absolute ABR latencies between 0 mg/kg/day (placebo), 1 mg/kg/day, or 2 mg/kg/day groups were found at 6 months of age, even though the iron supplementation resulted in a significant decrease in the prevalence of ID and IDA. Several possible explanations could be proposed. The duration or the severity were not at a magnitude high enough to result in prolonged ABR latencies, or these infants did not have a vulnerable phase of ABR maturation when ID occurred. Finally, ABR latencies could be affected later as a delayed effect of ID. Therefore, these results could suggest that ABR is not a sensitive indicator of impaired neurological development in MLBW infants or that severe ID does not cause such impairment at 6 months of age. In addition, a cord ferritin concentration is associated with ABR in premature infants. Cord ferritin lower than 75 ng/mL is associated with poor performance in motor skills and language development [62]. Iron status influences auditory neural maturation and infants with ID have a normal auditory neural maturation compared with a normal iron status [39] 

Berglund et al. [28] did not find any effect of iron supplementation on cognition at 3.5 years of age. Furthermore, marginally premature infants with low birth weight did not present a higher risk of cognitive problems compared with control infants, although the cognitive test performed was not accurate enough to predict later cognitive disabilities. Conversely, non-supplemented children have a significantly increased prevalence of behavioral problems; therefore, preventive iron supplementation is related to improved neurobehavioral development in infants at risk of ID. An iron intake of >1 mg/kg per day could be sufficient to decrease the risk of behavioral problems due to a correlation between iron intake and CBCL. Therefore, this study showed that iron supplementation reduced the risk of behavioral problems to a level similar to the controls. Many animal studies have reported that ID impairs brain development [28]. In addition, Berglund et al. [29] reported that this protective effect, which includes aggressive and rule-breaking behavior, persists until 7 years of age in children. 

Iron plays a crucial role in myelination, dendritic growth, and synaptogenesis. Therefore, ID could interfere with neurotransmitter function, mainly with regard to dopamine and other monoamines, which interestingly are closely associated with behavior [54]. Nonetheless, Berglund et al. [28] did not find a significant association between the behavioral outcome and iron status at 12 weeks or 6 months in infants. A possible explanation could be the lack of consensus biomarkers to define ID in infancy or because the used iron indicators lack specificity and sensitivity. Moreover, the iron availability in the CNS is not necessarily reflected by the biomarkers of iron status that reflect the iron status of peripheral blood and probably that of the bone marrow and the liver. In addition, Berglund et al. [29] revealed that the iron status at 6 months did not correlate with behavioral outcomes at either 3.5 or 7 years in LBW infants. This suggests that less iron is available for the brain tissue before ID can be detected in the blood by conventional haematological indices, since iron requirements for erythropoiesis are b prioritized over the brain iron supply [63]. 

Finally, Gupta et al. [25] evaluated neurodevelopment using a Developmental Assessment Scale for Indian Infants (DASII), a validated Indian adaptation of Bayley-II [64], in preterm infants who received iron supplementation as a standard. They did not find differences between infants who initiated complementary feeding at 4 months and those who continued with breast milk feeding and initiated complementary feeding at 6 months. The infants showed similar motor and mental development quotients. This study revealed that despite the fact that the infants received iron supplementation with either food or milk, their iron stores were greatly depleted.

## 5. Conclusions

In summary, the findings of this systematic review provide evidence that premature infants require iron supplementation in most of the cases and are susceptible to the development of ID or IDA, which could affect postnatal cognitive development and behavior. However, although ID impairs brain development due to its essential role in myelination, dendritic growth, synaptogenesis, and neurotransmitter function, motor, mental, and development parameters were not affected by iron supplementation in preterm neonates. Infants with iron depletion and overload showed a lower birth weight than those with a normal iron status. Taking into account these considerations, future research with more robust experimental designs and lower RoB are needed to achieve more solid conclusions.

## Figures and Tables

**Table 1:** Score breakdown on 8 items from the Delphi list for each randomized study.

Study	Items from the Delphi List									
	1	2	3	4	5	6	7	8	Total Score	
Gupta et al., 2017 [25]	1	1	1	NA	NA	NA	1	1	5	
Berglund et al., 2018 [29]	1	1	1	1	NA	1	1	1	7	These 4 references are all results from the same original cohort
Berglund et al., 2013 [28]										
Berglund et al., 2011 [27]										
Berglund et al., 2010 [26]										
Sankar et al., 2009 [24]	1	1	1	0	NA	NA	1	1	5	

Items of Delphi List: 1. Treatment allocation; 2. Were the groups similar at baseline regarding the most important prognostic indicators?; 3. Were the eligibility criteria specified?; 4. Was the outcome assessor blinded?; 5. Was the care provider blinded?; 6. Was the patient blinded?; 7. Were point estimates and measures of variability presented for the primary outcome measures?; 8. Did the analysis include an intention-to-treat analysis?; “yes” is indicated by a numeric score of 1 and “no” or “don’t know” is indicated by a 0; N/A: not applicable.

**Table 2:** Score breakdown on 22 items from the Transparent Reporting of Evaluations with Non-randomized Designs (TREND) Statement Checklist for each non-randomized study.

Study	Items from the TREND Statement Checklist																						
	1	2	3	4	5	6	7	8	9	10	11	12	13	14	15	16	17	18	19	20	21	22	Total Score
de Waal et al., 2017 [30]	1	1	0.75	N/A	1	0.66	0	0.33	1	0.5	0.25	0.85	0	0.25	0	0.5	0.5	0	0	0.33	1	1	10.92
Saha et al., 2016 [31]	1	1	0.5	N/A	1	0.66	1	0.66	0	0.5	0.75	0.85	1	0.5	0	1	0	1	0	0.33	1	1	13.75
Akkermans et al., 2016 [32]	1	1	1	N/A	1	0.66	0	0.33	0	0.5	0.75	0.71	0	0.5	0	0.5	0.5	1	0	0.33	1	1	11.78
Uijterschout et al., 2015 [33]	0.66	1	0.5	N/A	1	0.66	1	0.33	0	0.5	0.75	0.85	0	0.25	0	1	1	1	0	0.33	1	1	12.83
Yamada and Leone 2014 [34]	1	1	1	N/A	1	0.33	1	0.66	0	0.5	0.5	0.85	1	0.25	0	0.5	0.5	0	0	0.66	1	1	12.75
Mukhopadhyay et al., 2012 [35]	1	1	0.75	N/A	1	0.66	1	0	1	0.5	0.75	0.28	0	0.25	0	1	1	1	0	0.66	1	1	13.85
Amin et al., 2012 [36]	1	1	0.75	N/A	1	0.66	0	0.66	0	0.5	0.5	0.71	1	0.5	0	0.5	0.5	0	0	0.66	1	1	11.94
Kitajima et al., 2011 [37]	0.66	0.5	0.75	N/A	1	0.33	0	0	0	0	0.66	1	1	0.5	1	1	0.33	0	0	0.66	1	1	11.39
Molloy et al., 2009 [38]	0.33	1	0.75	N/A	1	0.66	1	0.66	0	0.5	0.5	0.85	1	0.5	0	1	0.33	0	0	0.33	1	1	12.41
Amin et al., 2010 [39]	1	1	0.75	N/A	1	0.33	0	0.33	0	0.5	0.5	0.71	0	0.5	0	1	0.33	0	0	0.33	1	1	10.28

N/A: not applicable.

**Table 3:** Summary of the characteristics of the randomized studies. LBW, low birth weight; CBCL, Child Behavior Checklist; ABR, auditory brainstem response; ID, iron deficiency.

Authors, Years	Study Design	Randomized	Sample Size	Male-Female	Preterm Weeks Age	Iron Supplementation	Dosage mg/kg/day	Key Findings
Berglund et al., 2018 [29]	controlled, double-blind, interventional trial	Yes	285	179–106	<37	Yes	1 or 2	Early iron supplementation in LBW infants prevents behavioral problems at school age, recommending iron supplementation in this population.
Gupta et al., 2017 [25]	open-label, multicentre trial	Yes	401	213–188	<34	Yes	2–3	There were no significant differences in motor and mental development quotients in premature infants who received complementary feeding at 4 months, or continuation of milk feeding and initiation of complementary feeding at 6 months.
Berglund et al., 2013 [28]	controlled, double-blind, interventional trial	Yes	319	158–161	<37	Yes	1 or 2	Iron supplementation reduced the prevalence of behavioral problems, defined as abnormal CBCL scores. Marginally low birth weight infants should be included in iron supplementation programs during early infancy.
Berglund et al., 2011 [27]	controlled, double blinded intervention trial	Yes	223	109–114	<37	Yes	1 or 2	Iron supplementation did not improve ABR latencies, and iron-deficient marginally low birth weight infants did not have impaired ABR latencies at 6 months. ABR is not a sensitive measure of impaired neurological development or that mild/moderate ID causes no such impairment in these infants.
Berglund et al., 2010 [26]	controlled, double-blind, interventional trial	Yes	285	138–147	<37	Yes	1 or 2	Iron supplementation from 6 weeks to 6 months reduces this risk of ID and IDA effectively, with no short-term adverse effects on morbidity or growth.
Sankar et al., 2009 [24]	Observacional	Yes	46	24–22	33	Yes	3–4	Supplementing iron at 2 weeks of life did not improve either serum ferritin or haematological parameters at 2 months of age in preterm very low birth weight infants.

**Table 4:** Summary of characteristics of non- randomized studies. ZnPP/H, Zinc protoporphyrin/heme; SF, serum ferritin.

Authors, Years	Study Design	Randomized	Sample Size	Men-Women	Preterm Weeks Age	Iron Supplementation	Dosage mg/kg/day	Key Findings
de Waal et al., 2017 [30]	prospective cohort study	No	161	98–63	32–36	No	–	ZnPP/H can be of additional value to detect infants at risk for IDA
Saha et al., 2016 [31]	prospective observational study	No	67	32–35	36.8–37.3	No	–	Late preterm and term small gestational age infants have adequate iron stores at birth and at 2 months of age
Akkermans et al., 2016 [32]	prospective multi-centre study	No	68	43–25	32–35	No	–	Iron depletion is common in late preterm infants at the age of 6 weeks in a setting without standardized iron supplementation. Early iron supplementation should be considered in late preterm infants with a low birth weight or low SF in the first week of life.
Uijterschout et al., 2015 [33]	prospective cohort study	No	143	87–56	32–36	No	–	Preterm infants have an increased risk of ID compared with those born at term. Supporting the need of iron supplementation. Measurement of ferritin at the age of 1 week might be useful to identify those infants at particular risk.
Yamada and Leone 2014 [34]	Cohort study	No	46	27–19	34–36	Yes	2	Exclusively breastfed late-preterm newborns presented greater reductions in hemoglobin/hematocrit and lower iron stores than term newborns. Specific iron supplementation is suggested.
Mukhopadhyay et al., 2012 [35]	prospective cohort study	No	150	47–103	≤36	No	–	Preterm small gestational age infants have lesser total body iron stores as compared to preterm adequate gestational age infants at birth. Similarly preterm infants have less iron stores than term infants.
Amin et al., 2012 [36]	prospective observational study	No	131	67–64	24–32	Yes	2	Iron parameters at 35 weeks post-menstrual age is extremely variable and is predicted by erythrocyte transfusions. Due to the harmful effects of iron overload and latent iron deficiency status, iron homeostasis maintenance is crucial in the neonatal period.
Kitajima et al., 2011 [37]	prospective cohort study	No	71	29–32	<37	No	-	Preterm infants have lower prohepcidin production at birth according to the gestational age, and the levels might be susceptible to the in utero stress. The postnatal increase might reflect the maturation and/or adaptation of iron homeostasis.
Amin et al., 2010 [39]	prospective cohort study	No	80	41–39	27–33	No	-	Premature infants with iron deficiency have abnormal auditory neural maturation compared with infants with normal iron status.
Molloy et al., 2009 [38]	Observational	No	60	22–38	26.5	Yes	2–4	Careful evaluation of iron indices is essential to prevent potential organ injury and unnecessary iron supplementation which could induce iron overload.
